# A Narrative Review on the Potential of Red Beetroot as an Adjuvant Strategy to Counter Fatigue in Children with Cancer

**DOI:** 10.3390/nu11123003

**Published:** 2019-12-07

**Authors:** Maria C. Swartz, Kaitlyn Allen, Rachel R. Deer, Elizabeth J. Lyons, Michael D. Swartz, Tom Clifford

**Affiliations:** 1 Department of Pediatrics-Research, The University of Texas MD Anderson Cancer Center, 1515 Holcombe Blvd., Houston, TX 77030-4009, USA; 2 Department of Nutrition and Metabolism, The University of Texas Medical Branch, 301 University Blvd., Galveston, TX 77555-1124, USA; ksallen@utmb.edu (K.A.); ellyons@utmb.edu (E.J.L.); 3 Division of Rehabilitation Sciences, The University of Texas Medical Branch, 301 University Blvd., Galveston, TX 77555-1137, USA; rrdeer@utmb.edu; 4 Department of Biostatistics and Data Science, The University of Texas Health Science Center, School of Public Health, 1200 Pressler St., Houston, TX 77030, USA; Michael.d.swartz@uth.tmc.edu; 5 School of Sport, Exercise and Health Sciences, Loughborough University, Leicestershire LE11 3TU, UK; t.clifford@lboro.ac.uk; 6 School of Biomedical, Nutritional and Sport Sciences, Newcastle University, Newcastle NE2 4HH, UK

**Keywords:** neoplasms, beetroot, betalains, exercise, childhood cancer, pediatric cancer, fatigue, nutrition

## Abstract

Cancer-related fatigue (CRF) is a debilitating adverse effect among children with cancer and a significant barrier to physical activity (PA) participation. PA interventions are effective at reducing fatigue and improving both quality of life (QOL) and functional outcomes in children with cancer. However, 50–70% of children with cancer do not meet PA guidelines. Thus, adjuvant methods are needed to increase PA participation. Given the growing interest in the use of beetroot juice to reduce exercise-induced fatigue, our narrative review evaluated the potential use of beetroot to improve PA participation to counter CRF and improve QOL. Our review of 249 articles showed a lack of published clinical trials of beetroot in children and adults with cancer. Trials of beetroot use had been conducted in a noncancer population (*n* = 198), and anticancer studies were primarily in the preclinical phase (*n* = 40). Although results are promising, with beetroot juice shown to counter exercise-induced fatigue in a variety of athletic and patient populations, its use to counter CRF in children with cancer is inconclusive. Pilot and feasibility studies are needed to examine the potential benefits of beetroot to counter CRF, increase PA participation, and improve QOL in children with cancer.

## 1. Introduction

### 1.1. Cancer-Related Fatigue and Its Impact on Pediatric Cancer Patients

Childhood cancer is the second leading cause of death among children aged 1 to 14 years in the United States [1]. Based on the latest estimate, approximately 10,590 children (<15 years old) will be diagnosed as having cancer in the United States [1]. With advanced diagnosis and treatment, the 5-year survival rate is now more than 80% in children [2]. However, cancer treatment is associated with a multitude of adverse effects [3,4]. Cancer-related fatigue (CRF) is one of the most significant and debilitating problems associated with cancer treatment [3,5] and often persists even after treatment has ended [6]. CRF is defined as distressing, pervasive symptoms with physical, mental, and emotional components characterized by a lack of energy [5,7]. Children describe CRF in terms of physical sensations such as tiredness, sleepiness, lack of energy, and lack of desire to interact and participate in daily activities [5]. In addition, an increase in fatigue has been associated with poor sleep quality [8], a decrease in sleep duration [9,10], sleep disturbances [11], and decreased physical performance [12]. Furthermore, CRF has been associated with depression [5,13] and poor neurocognitive outcomes represented by poorer performance in task efficiency and memory tests [14]. Thus, CRF ultimately affects the quality of life (QOL) of children with cancer by impeding patients’ ability to engage in daily activities [15]. As a result, there is a growing interest in developing non-pharmacological interventions to minimize fatigue and its negative impact on pediatric cancer patients [16,17].

### 1.2. Critical Role of Physical Activity among Children with Cancer

Multiple non-pharmacological interventions have been developed to reduce fatigue in children with cancer [16,17]. These include exercise, massage, healing touch, music therapy, acupressure, and health education [16,17]. Reviews of the literature have found that physical activity (PA) is the most frequent target for intervention [16,17]. PA is defined as the movement of the body supported by the skeletal muscle with energy expenditure. This differs from the term exercise. Exercise is a subcategory of PA with the goal of improving one or more areas of physical fitness by following a planned, structured, and repetitive exercise program [18]. Given that children with cancer spend more time engaging in sedentary behavior (e.g., screen-time) than do children without cancer, promoting engagement of any type of PA is critical to help maintain physical function [19]. For example, a nonrandomized trial showed that supervised aerobic and resistance exercise sessions during treatment were able to significantly improve functional capacity and body mass in children who were undergoing hematopoietic stem cell transplantation [20]. 

Furthermore, systematic reviews with and without meta-analyses of randomized controlled trials indicated that PA interventions significantly decreased fatigue and stress [16] and produced a large effect size of −0.76 in reducing general fatigue in children with cancer [21]. Researchers used various exercise interventions of lengths ranging from two days to 16 weeks [16,21]. Most of the studies focused on promoting aerobic exercise training [16,21]. Aerobic exercises were implemented with the use of compact discs for home-based interventions, bicycle-style exercisers in the clinic, or supervised training sessions [16,21]. The target exercise intensity from interventions ranged from 60% to 90% of maximum heart rate within a 25- to 45-minute exercise session [21]. Across reviews, only two studies [22,23] included a core strength and flexibility training component. Although limited by small sample sizes and various types of exercise interventions, the aforementioned studies showed that PA (both aerobic and resistance exercises) plays a critical role in mitigating fatigue and in improving function during and after active cancer treatment in children with cancer. 

Despite evidence showing that PA in children with cancer during treatment is safe and has positive benefits (i.e., decreased fatigue, improved physical fitness and motor function, and improved QOL) [24,25], 50–70% of children with cancer do not meet the PA guidelines of 60 min of aerobic exercise and muscle strengthening or bone-strengthening activities per day [26]. Evaluation of PA levels using accelerometers confirmed that children with acute lymphoblastic leukemia had significantly lower levels of total weekly moderate-to-vigorous physical activity (328 ± 107 min) than did control children (506 ± 175 min) [27]. Furthermore, a recent meta-analysis of observational studies showed that children with cancer had significantly lower levels of PA (g = −0.889) and fitness (g = −1.435) than did controls without cancer [28]. Additionally, the adherence rate of children with cancer to PA interventions ranged widely, from 25% to 100% [25]. Therefore, innovative methods are needed to promote PA initiation and adherence among childhood cancer survivors. 

### 1.3. Benefits and Risks of Dietary Nitrate 

One possible method to augment PA participation may be the use of beetroot juice as a supplement during cancer treatment. Researchers have found that beetroot has been used as an alternative medicine in cancer patients around the globe [29]. This is because beetroot has been viewed as a “medicinal food” since ancient times [29]. Nikolic et al. found that more than half (~57%) of gastrointestinal cancer patients (*n* = 193) reported using beetroot juice in conjunction with conventional oncology treatment [30]. Arrko et al. found that 45% of Finnish cancer patients (*n* = 151) have used a variety of unproven cancer remedies; among these patients, approximately 10.3% have tried or were using beetroot as an alternative remedy [31]. In addition, Clement et al. looked at the most commonly used herbal remedies and functional foods among prostate, breast, and colorectal cancer patients (*n* = 150) in Trinidad [32] and found that 19.3% of the patients used beetroot, either juiced or blended, during treatment. In addition, they found that 47.3% of patients believed that supplementation of beetroot during treatment was beneficial in managing their cancer diagnosis. Furthermore, there is growing evidence of health benefits from the high natural nitrate content in beetroot juice [33]. Some of these benefits include enhanced exercise performance, increased exercise tolerance, reduced exercise-related fatigue, inhibited cancer proliferation, and reduced adverse effects associated with anthracyclines [29,33,34,35,36]. This is because dietary nitrate from vegetables, such as beetroot, is an important source of nitric oxide (NO) in the nitrate–nitrite–NO pathway [37]. Researchers have found that it is through this pathway that natural nitrate compounds have a positive impact on health [37,38]. 

Nitrate (NO3-)—a compound that naturally occurs in vegetables—has been used as a preservative, specifically in processed meats [37,39]. Through the interaction with bacteria in the mouth or enzymes in the body, nitrates are then turned into nitrites (NO2-). Nitrites can then become NO or nitrosamines [39]. Nitrosamines are a known carcinogen associated with increased bowel cancer; they occur when nitrites combine with a source of amines (e.g., protein-rich foods) [37,38,40]. Nitrosamines can also occur when meats are cooked with high heat. In contrast, nitrites formed from vegetable sources have been found to encourage NO formation and inhibit nitrosamine formation [37,40]. This is because nitrites from vegetable sources are combined with protective components like vitamin C, polyphenols, and fiber that promote NO formation. Thus, the World Health Organization’s International Agency for Research on Cancer (IARC) concluded in 2006 that dietary nitrate consumed without the presence of antioxidants is probably carcinogenic [37,40]. Furthermore, although dietary nitrates from vegetable sources are now considered to be safe [37,38,41], excessive levels of nitrates (>10 mg/L) from drinking water have been associated with methemoglobinemia in infants (<6 months old) and in those whose conditions inhibit the conversion of methemoglobin to hemoglobin [42]. However, such excessive consumption of dietary nitrate through food sources is hard to achieve unless the drinking water has been contaminated with fertilizers in the groundwater [42]. 

## 2. Methods 

Similar to the review completed by Kapadia in 2012 [29], our literature search was conducted with the assistance of a research librarian. It yielded no clinical trials that evaluated the use of beetroot juice in children with cancer (Table 1). The search terms that we used included beet, sugar beet, beta vulgaris, betanin neoplasm/cancer, pediatrics/paediatric, adolescent, endurance, fatigue, athletic performance, and stamina. Databases that the librarian searched included Embase, Medline, and Cochrane. Articles were first screened by title, abstract, and whether they were written in the English-language by authors MCS and KA. The full-text of the articles was pulled for review if outcomes of interest corresponded with our research question. Additional articles were identified by examining reference lists and from expert input. Of the 249 articles that were selected for review, all 198 clinical trials associated with the use of beetroot juice had been conducted in a noncancer population, and the 40 anticancer studies were primarily in the preclinical phase. The remaining 11 studies consisted of a combination of review articles and mechanistic studies associated with the use of beetroot to increase exercise endurance and within various disease conditions using animal and cell models. Therefore, the goal of our narrative review was to compile evidence from studies examining the effects of beetroot on chemoprevention and exercise-induced fatigue to evaluate its potential to combat CRF, increase PA participation and improve QOL in children with cancer. 

## 3. Current Research on Beetroot and Cancer 

In our review of the literature on beetroot use in cancer research, we found that the focus has been primarily on evaluating beetroot’s potential chemopreventive and anticancer effects using cancer cell lines [29,35]. Briefly, the chemopreventive and anticancer effects of beetroot and its compounds occur through several mechanisms such as: inducing cell apoptosis, reducing oxidase activity, disrupting inflammation, increasing anti-inflammatory cytokines, and acting as a cytotoxin [29,35]. Studies within the past decade have found that beetroot was able to suppress the growth of breast cancer cells (MCF-7) in a dose-dependent manner [43], that fermented beetroot juice was able to decrease the average number of aberrant crypt foci in rat colons [44], that a beet-rich diet was able to decrease the incidence and amount of aberrant crypt foci in male rats [45], and that the addition of beetroot extract resulted in a slower rate of proliferation of esophageal papilloma in rats [35]. These studies used a wide range of dosages in cell and animal studies to examine the various beetroot compounds’ anticancer functions. At this time, additional research is needed to determine clinically relevant doses of beetroot that can be used in humans. 

Other research with cell lines has examined beetroot as an adjuvant to anthracyclines chemotherapy. Anthracyclines (such as doxorubicin) are a class of chemotherapy drugs that are commonly used to treat many types of cancer, yet are also well known to cause cardiotoxicity [46]. Researchers theorized that since betanin (a beetroot compound) has a chemical structure similar to that of doxorubicin, beetroot might exhibit similar cytotoxic effects on cancer cells [47], and adjuvant therapy might lead to a reduced dosage of doxorubicin, thus reducing cardiotoxicity. Kapadia et al. found that in prostate cancer (PC-3) and breast cancer (MCF-7) cell lines, red beetroot extract exhibited dose-dependent cytotoxic effects similar to those of doxorubicin, but overall, the cytotoxic effects of red beetroot extract were significantly lower [47]. Another group, Das et al., showed that after a combination of beetroot juice and doxorubicin was given to the adult rats, the beetroot reduced cardiotoxicity and cardiac cell deaths in the cardiomyocytes [34]. Furthermore, Das et al. found that using a combination of beetroot juice and doxorubicin, compared with use of doxorubicin alone, significantly increased doxorubicin-mediated apoptosis in breast cancer cells [34]. These important studies in cell lines and animal models highlight the possibility of using beetroot to treat cancer and attenuate cardiotoxicity and illustrate the potential usefulness of beetroot as an adjuvant therapy; however, they provide little information on the potential of beetroot to reduce cancer-related fatigue as a means to promote PA. Furthermore, given the potential interaction between beetroot supplementation and conventional neoadjuvant/adjuvant anticancer therapy, the safety of its use during treatment needs to be evaluated in future pilot trials among children and adults with cancer. 

## 4. The Use of Beetroot Juice in Cancer Patients

Our literature search revealed no published clinical trial results on beetroot used as a supplement to modify PA levels or reduce fatigue in adults or children with cancer. However, our search of Clinical Trials.gov revealed a total of five trials registered to evaluate the use of dietary beetroot in cancer patients. The outcomes being investigated include aerobic performance, cardiovascular function, muscle strength, and/or body composition and/or treatment-related symptoms. Table 2 provides a summary of the targeted population, outcomes of interest, and the type and dosages of beetroot supplements proposed by the studies. Currently, two studies are ongoing (one not yet recruiting [NCT03944226] and one enrolling [NCT03776149]), two had unknown status (NCT023193560; NCT02044562) and one study finished without achieving its recruitment goal (NCT02058849). The sole study (NCT02058849) reporting results compared a supplement of 10 g of beetroot powder (BEETELITE ™ NeO shot) mixed in 4–8 oz of water to placebo. Since the trial ended with only one person in the placebo group, a comparison between groups could not be conducted. 

As mentioned previously, despite the lack of clinical trials evaluating the effectiveness of beetroot juice in cancer patient populations, researchers have found that beetroot has been used as an alternative medicine in cancer patients around the globe [29]. Further, 67.4% of cancer patients stated that they were satisfied with their use of herbal remedies/functional foods, and 90% said they would continue to use the herbal remedies/functional foods in the long-term [32]. The top two reasons for using alternative medicine across all three studies were (1) patients believed it would be health-restoring and/or increase their chance of being cured [30,31,32] and (2) patients had heard about the benefits of this alternative medicine from other cancer patients [30,31,32]. The reports from Nikolic et al. [30], Arrko et al. [31], and Clement et al. [32] have all collected self-reported information on the use and benefits of beetroot, so they do not present rigorous evidence of the adverse events or benefits associated with beetroot supplements in cancer patients. Since beetroot is often used as an alternative medicine in conjunction with conventional anticancer therapy, feasibility and proof-of-concept pilot trials are needed to help formalize adverse events and establish the potential benefits of beetroot supplements on various cancer and treatment-related outcomes such as those presented in Table 2. 

## 5. Effects of Beetroot on Exercise Tolerance in Athletes and Disease Populations 

Given the sparsity of evidence in the literature describing beetroot’s effect on cancer-related fatigue, we compiled evidence of beetroot’s ability to reduce fatigue in athletes and disease populations to illustrate beetroot’s potential feasibility to intervene with cancer-related fatigue.

### 5.1. Exercise Tolerance in Athletes

In the past decade, interest has been growing in the effects of beetroot juice on exercise tolerance and fatigue. This interest initially stemmed from a study by Larsen et al., who found that dietary nitrate, which beetroot contains in abundance, reduces—presumably via conversion to nitric oxide (NO)—the O_2_ cost of submaximal exercise, thereby improving muscular efficiency [48]. Because efficiency is a key determinant of aerobic exercise performance, it was reasonable to assume that this nitrate-induced reduction in work demand could enhance performance by increasing exercise tolerance and delaying the onset of fatigue [41]. 

Although nitrate is not believed to possess any specific physiological function, its conversion to the free radical NO can mediate a multitude of beneficial effects. As previously mentioned, the production of NO from nitrate starts in the small intestine, where the highly bioavailable nitrate is absorbed into the circulation [49]. Approximately 25% of the nitrate is taken up by the enterosalivary cycle, where it can be reduced to nitrite [50]. The generation of NO occurs when the salivary nitrite is reabsorbed into the circulation via the stomach, where it is metabolized to NO by a variety of reductase enzymes [50]. The detailed processes involved are described elsewhere [50]. 

The first study to report the ergogenic potential of nitrate-rich beetroot was conducted by Bailey et al., who measured exercise efficiency in eight healthy male volunteers after they ingested beetroot juice (500 mL/day, nitrate; 11.2 mmol) or a nitrate-free, low-calorie, blackcurrant cordial (control) for 6 days [36]. They found that beetroot juice elicited a 5% reduction in whole-body O_2_ consumption (e.g., improved efficiency) during a sub-maximal cycling task equivalent to 80% of the pulmonary gas exchange threshold (GET). Furthermore, those randomized to the beetroot juice intervention group exhibited a 16% longer time to fatigue [36] compared with controls who underwent the same vigorous exercise activity [36]. These findings were confirmed by several further studies, in which beetroot juice was shown to extend the time to fatigue by 25% during high-intensity knee-extensor exercise [51]; by 15% during high-intensity running activity [52], and by 3.5% during intermittent cycling exercise [53]. Beetroot juice has also been shown to enhance peak power output during cycling [54] and maximal strength in response to electrical stimulation [55] and during voluntary muscle contractions [56]. 

Two of the aforementioned studies [52,53] and several others since (see [41] for review) have used a nitrate-depleted, but otherwise nutritionally identical, beetroot juice drink as a placebo, suggesting that nitrate was the bioactive constituent largely responsible for these beneficial physiological effects. However, recent research has shown that betalains, the group of pigments found in beetroot that provide its purple hue, can also increase exercise tolerance in healthy young subjects, indicating that nitrate might not be the only ingredient in beetroot with ergogenic effects [57,58]. Although the mechanisms underpinning such improvements are unclear, betalains are known to have potent antioxidant effects [33]. Such effects are believed to attenuate exercise-induced oxidative stress, which has been associated with fatigue during high-intensity exercise [59]. Thus, the betalains present in beetroot could enhance exercise performance independent of any physiological effects of nitrate and its subsequent conversion to NO [58,59,60]. Finally, a study by Porcelli et al. found that a short-term high-nitrate diet (~8.2 mmol/day of nitrate) significantly improved exercise performance compared with performance associated with the control diet (~2.9 mmol/day of nitrate) [61], suggesting that future studies are needed to examine the effects of beetroot juice on exercise performance compared with the effects of a high-nitrate diet from other vegetable sources. 

### 5.2. Exercise Tolerance in Populations with Various Disease Conditions

Far fewer studies have investigated whether beetroot supplementation can improve exercise tolerance and reduce fatigue in populations with various disease conditions (see [62] for a review). Of those that have, most studies of beetroot’s effects on health have focused on persons with cardiovascular disease (CVD), who typically have a reduced aerobic capacity, exercise intolerance, and an early onset of fatigue during both physical activity and daily living tasks [63]. In one of the first of these studies, Kenjale et al. investigated the influence of a pre-exercise dose of beetroot juice (500 mL) on exercise tolerance in patients with peripheral arterial disease [64]. They showed that beetroot juice significantly extended time to exhaustion (+ 18%) during a controlled treadmill-walking task in comparison to a placebo (orange juice). They also reported increases in total hemoglobin concentration in the gastrocnemius muscle, as measured by near-infrared spectroscopy, suggesting that beetroot juice might have stimulated peripheral vasodilation and tissue oxygenation in the ischemic region [64]. More recently, Coggan et al., reported that ingesting beetroot juice before sub-maximal cycling exercise extended time to fatigue by 7% compared to a placebo in heart-failure patients [65]. 

As for other disease conditions, a recent study also found that beetroot juice improved exercise tolerance in obese adolescents, another population with poor aerobic capacity and exercise intolerance. This study found that beetroot juice could be used to combat the early onset of fatigue associated with exercise in this population [66]. Similarly, Berry et al. and Curtis et al. found that beetroot juice supplementation in individuals with chronic obstructive pulmonary disease (COPD) resulted in extended exercise time during submaximal exercise, decreased resting systolic blood pressure, lowered VO2 consumption during submaximal exercise, and decreased diastolic blood pressure [67,68]. The favorable effects of beetroot juice do not appear to be limited to aerobic exercise; benefits for muscle strength have also been reported. In one such study, in an elderly population, de Oliviera et al. found that acute intake of a beetroot-based gel attenuated the decline in handgrip strength following fatiguing handgrip exercise, indicating that beetroot juice might also be useful for counteracting the decline in muscle strength and power associated with age [69,70].

It is important to note that not all studies report favorable effects with beetroot juice, both in healthy populations and in those with various disease conditions [62]. In the latter population, several studies found no differences in exercise tolerance from beetroot juice ingestion in patients with CVD [71,72] or type 2 diabetes [73,74] or individuals with COPD [75,76,77]. The equivocal findings in studies to date are believed to be due to differences in the nitrate dose used, exercise mode, populations, and/or potential interference from concomitant medications [62]. Nevertheless, a meta-analysis of studies conducted in healthy populations suggested that dietary nitrate (beetroot juice or sodium/potassium nitrate) does have a small to moderate beneficial effect on exercise performance [78]. When coupled with studies showing positive effects in various disease populations, it appears that beetroot juice does hold promise as a dietary supplement to combat exercise intolerance and fatigue and warrants further research. 

### 5.3. Mechanisms of Exercise Tolerance Effect 

The mechanisms by which beetroot enhances exercise tolerance are not well-understood. One potential explanation, presented by Bailey et al., is that nitrate, via its conversion to NO, reduces the adenosine triphosphate (ATP) cost of muscle contraction, possibly by downregulating the energy required for intramuscular calcium handling (i.e., actomyosin-ATPase and Ca^2+^-ATPase activity) [36,51]. There is also convincing evidence that the ergogenic effects of beetroot juices are mediated by nitrate-induced structural alterations in the mitochondria. This was demonstrated by Larsen et al., who obtained muscle biopsies from 14 healthy participants after 3 days of sodium nitrate supplementation (0.1 mmol kg^-1^, day^-1^) or a placebo control [79]. They found that nitrate supplementation decreased the mitochondrial protein content of two transporter proteins involved in ATP/adenosine diphosphate (ADP) proton uncoupling during hydrolysis: adenine nucleotide translocase (ANT) and mitochondrial uncoupling protein 3 (UCP-3). This decrease translated to reduced energy (ATP) loss during ATP hydrolysis and was reflected by a 23% increase in ATP production. The net result was a 19% improvement in the oxidative phosphorylation ratio, or in other words, a more efficient use of O_2_ to produce energy during muscular contraction [79]. 

Alternatively, a number of studies provided evidence that adding nitrate-rich beetroot juice (1 mmol kg^-1^, day^-1^) to a rat’s diet for 5 days augments type IIb fiber-specific blood flow during submaximal running exercise [80,81]. Therefore, it cannot be ruled out that the ergogenic effects of nitrates are, at least in part, due to improved local perfusion and muscle oxygenation in more anaerobic muscle fibers, thereby reducing muscle metabolic perturbation (i.e., Phosphocreatine depletion, blood lactate accumulation) during exercise [41]. Of course, these effects might operate in tandem or perhaps are even cumulative [82]. For a more detailed review of the mechanisms of beetroot’s ergogenic potential see the review conducted by Jones et al. [41]. 

## 6. Conclusions and Future Directions

The goal of this narrative review was to provide an overview of the use of beetroot juice in cancer in the context of previous systematic reviews. Despite apparent widespread use among patients as complementary medicine, beetroot juice has not been widely studied in this context. Trials registered in ClinicalTrials.gov showed that researchers are beginning to evaluate the potential use of dietary beetroot juice in adult cancer patients to improve aerobic performance, cardiovascular function, muscle strength, body composition, and/or decrease treatment-related symptoms, but this research has not yet been published to our knowledge. Through our brief review of preclinical evidence and from clinical trial evidence in athlete and diseased populations, beetroot and its constituents appear to hold promise to counter exercise intolerance and fatigue, but its use for such a purpose in children with cancer remains inconclusive and needs to be examined in proof-of-concept pilot studies. Thus, our review points to a new direction in which beetroot has the potential to be used as an adjuvant strategy to improve PA participation to counter CRF in children with cancer.

Further, our findings suggest that pilot and feasibility trials will be a critical next step. Information on safety and potential adverse effects is needed due to the widespread use of beetroot juice as a supplement in cancer patients. Currently, there is no clear evidence of adverse events associated with beetroot supplementation or of the benefits of beetroot supplements in cancer patients. Given that beetroot and its constituents have been shown to mitigate some of the negative effects of cancer on cell lines and to counter exercise-induced fatigue in young and diseased populations, there is a rationale to examine its potential benefits for reducing CRF in children with cancer, in a manner similar to that in the ongoing trials in adults with cancer. More research is needed to (1) delineate the anticancer and CRF reduction mechanism(s) of action in tandem with traditional anticancer treatment, (2) move from preclinical testing to pilot and feasibility studies to formally evaluate the safety and potential efficacy of beetroot juice supplementation in the pediatric cancer patient population to reduce fatigue during exercise, and (3) assess the potential synergistic effect of beetroot with standard anticancer drugs to potentially reduce their adverse effects. 

## Figures and Tables

**Table 1:** Search terms used for the narrative review.

1	exp beet/ or exp sugar beet/	3268
2	(beet * or “Beta vulgaris” or Betanin).ab,ti.	18,668
3	1 or 2	19,159
4	exp neoplasm/	4,623,242
5	(cancer * or neoplasm * or leuk * or lymphoma *).ab,ti.	3,205,201
6	4 or 5	5,412,111
7	3 and 6 [broad, beets and cancer]	482
8	exp athlete/	53,800
9	exp performance/	32,549
10	exp endurance/	23,154
11	(athlete * or performance * or endurance * or cardio *).ab,ti.	2,218,686
12	8 or 9 or 10 or 11	2,240,627
13	3 and 12 [beets and athletic performance and stamina]	1180
14	exp pediatrics/	113,020
15	(child * or pediatric * or paediatric * or adolescen * or infant *).ab,ti.	2,520,664
16	14 or 15	2,544,678
17	3 and 16 [beets and pediatric population]	213
18	7 or 13 or 17	1809
19	Beetle.ab, ti.	18,518
20	18 not 19	1312

**Table 2:** Registered trials in Clinical Trials.gov investigating beetroot supplement in cancer patients.

NCT Trials	Cancer Population	Outcomes Investigated	Duration	Dosage	Brand
03944226	Breast Cancer	Lipid composition in tumor and breast tissue; water displacement in tumor	5-day dietary nitrate intervention	3 doses of 7 cL/70 mL (0.4 g nitrate/dose) of concentrated beetroot juice/day	James White Drinks, UK
03776149	Cancer Survivor with History of Anthracycline Chemotherapy	Left ventricular function	7-day dietary nitrate (beetroot juice)	140 mL/day	Beet It (Heart-Beet Ltd.)
02058849	Head and Neck Cancer	Adherence and endurance at 6 weeks and 12 weeks	Up to 6 weeks	10 g beetroot crystals mixed with 4–8 oz of water/day	BEETELITE™ NeO shot
02319356	Nasopharyngeal Carcinoma	Anaerobic threshold; VO2Max	7-day dietary nitrate	NO3- 6.2 mmol/day (500 mL)	Beet It Sport Shots, James White Drinks, UK
02044562	Colorectal Cancer	Plasma nitrate level	7-day dietary nitrate	70 mL/day (0.45 g nitrate)	Did not report
